# Prenatal maternal inactivated COVID-19 vaccination: the maternal and neonatal outcomes, a retrospective cohort study

**DOI:** 10.3389/fphar.2024.1299213

**Published:** 2024-02-28

**Authors:** Yaoyao Cai, Shenghao Wu, Sisi Zhang, Xinxin Xu, Fengfeng Xie, Lijun Gao, Weiting Xia

**Affiliations:** ^1^ Department of Obstetrics, The First Afffliated Hospital of Wenzhou Medical University, Wenzhou, China; ^2^ Reproductive Medicine Center, Department of Obstetrics and Gynecology, The Second Affiliated Hospital and Yuying Children’s Hospital of Wenzhou Medical University, Wenzhou, China; ^3^ Reproductive Medicine Center, Department of Obstetrics and Gynecology, The First Affiliated Hospital of Wenzhou Medical University, Wenzhou, China; ^4^ Department of Gynaecology, Women’s Hospital, Zhejiang University School of Medicine, Hangzhou, China; ^5^ Department of Gynecology, The First Affiliated Hospital of Wenzhou Medical University, Wenzhou, China

**Keywords:** COVID-19, inactivated vaccine, maternal outcomes, pregnancy, neonatal outcomes

## Abstract

**Background:** Despite the widespread adoption of COVID-19 vaccination, a comprehensive understanding of potential vaccine-induced adverse effects, particularly in the context of pregnancy, remains a critical area of investigation. Elevated concerns surround the maternal and neonatal outcomes subsequent to prenatal maternal COVID-19 vaccination. While existing studies have provided insights into the safety profile of COVID-19 mRNA vaccines, the extrapolation of these conclusions to inactivated COVID-19 vaccines poses uncertainties. Notably, limited data are available regarding the maternal and neonatal effects associated with inactivated COVID-19 vaccines.

**Objective:** To evaluate the prenatal maternal inactivated COVID-19 vaccination and the impact on maternal and neonatal outcomes.

**Methods:** This was a retrospective cohort study of women who delivered between January and June 2022 at a single university-affiliated hospital. Those who have completed at least one dose of inactivated vaccine before or during pregnancy were included in “vaccinated group,” and those who were not vaccinated were included in “unvaccinated group,” the maternal, pregnancy and neonatal outcomes were evaluated. Propensity score matching (PSM) was performed to balance the baseline parameters of the two groups.

**Results:** A total of 1926 women were enrolled in this study, 827 (42.94%) women were prenatally vaccinated, and 1099 (57.06%) unvaccinated. The gestational week of delivery were slightly lower in the vaccinated group, 38.61 ± 1.89 weeks in the vaccinated group and 38.93 ± 1.49 weeks in the unvaccinated group. There was a higher rate of overall preterm delivery in the vaccinated group (aOR 1.61, 95% CI 1.07–2.42; *p* = 0.02), however, the probability of delivery before 34 weeks and before 32 weeks (early preterm delivery) were similar (*p* > 0.05). A total of 2009 infants were born, 851 in the vaccinated group and 1158 in the unvaccinated group. There were similar neonatal outcomes in the two groups.

**Conclusion:** Although we found a slightly lower gestational week of delivery and a possible increased rate of late preterm birth in the vaccination group, there was no difference in mean neonatal weight, incidence of low birth weight infants and other neonatal adverse complications. Meanwhile, there was no difference in pregnancy and maternal outcomes between the two groups.

## Introduction

Over the last 3 years, the COVID-19 outbreaks and pandemics have caused catastrophic damage to health, economy, and all aspects of the society worldwide. Reported to WHO, there have been 754,018,841 confirmed cases of COVID-19, including 6,817,478 deaths (World Health Organization, 2023). To date, mass vaccination presents a vital strategy to alleviate illness and establish herd immunity for controlling the pandemic. As of 30th January 2023, a total of 13,168,935,724 vaccine doses have been administered (World Health Organization, 2023). Among them, COVID-19 mRNA vaccine (BNT162b2) has become the world’s first COVID-19 vaccine authorized by the FDA, due to its advantages such as a quick development cycle, no requirement for cell culture, and high immunogenicity. It has been reported that more than 85% of the risk of symptomatic disease and its transmission can be reduced by vaccination (Dagan et al., 2021). However, the need to store at ultra-low temperatures could pose a challenge when transporting and storing mRNA vaccines. However, inactivated COVID-19 vaccines have the advantage of being easily stored and shipped at 2°C–8°C for years, and are predominantly used in China. Based on a randomized clinical trial, the efficacy of inactivated COVID-19 vaccines was shown to be over 70% for the prevention of symptomatic COVID-19 (Al Kaabi et al., 2021). Despite the high coverage of COVID-19 vaccination, the potential for vaccine-induced adverse effects has not been fully elucidated, particularly in pregnant women who did not participate in the preliminary randomized controlled trial of COVID-19 vaccine safety and efficacy. This paucity underscores the imperative for further rigorous research to delineate the specificities of vaccine safety profiles, particularly in the intricate context of pregnancy.

According to a meta-analysis, there was no evidence of an increased risk of adverse perinatal outcomes after COVID-19 mRNA vaccination in pregnancy, but rather a risk of reduced stillbirth rate (Prasad et al., 2022). Meanwhile, as a multicentre retrospective cohort study shown, vaccination of the COVID-19 mRNA vaccines during the third trimester of pregnancy was not associated with adverse maternal outcomes, moreover, it decreased the risk for neonatal adverse outcomes (Rottenstreich et al., 2022). Similar results were found in another retrospective cohort study, which demonstrated no differences in pregnancy, delivery and neonatal complications (including gestational age at delivery, small for gestational age and the incidence of neonatal respiratory complications) with or without COVID-19 mRNA vaccination (Goldshtein et al., 2022).

In China, inactivated COVID-19 vaccines are widely promoted. Diffierent with the mRNA vaccines described above, these inactivated vaccines were developed based on the immune response induced by inactivated SARS-CoV-2 virus. There are limited data on the maternal and neonatal effects of inactivated COVID-19 vaccines. Therefore, more relevant data are needed to inform the outcome of mothers, pregnancies and infants. Such insights are paramount for informing precise and evidence-based recommendations for prenatal maternal inactivated COVID-19 vaccination. Our research aims to evaluate the impact of prenatal maternal vaccination of the inactivated COVID-19 vaccination regarding maternal and neonatal adverse outcomes.

## Materials and methods

**FIGURE 1:**
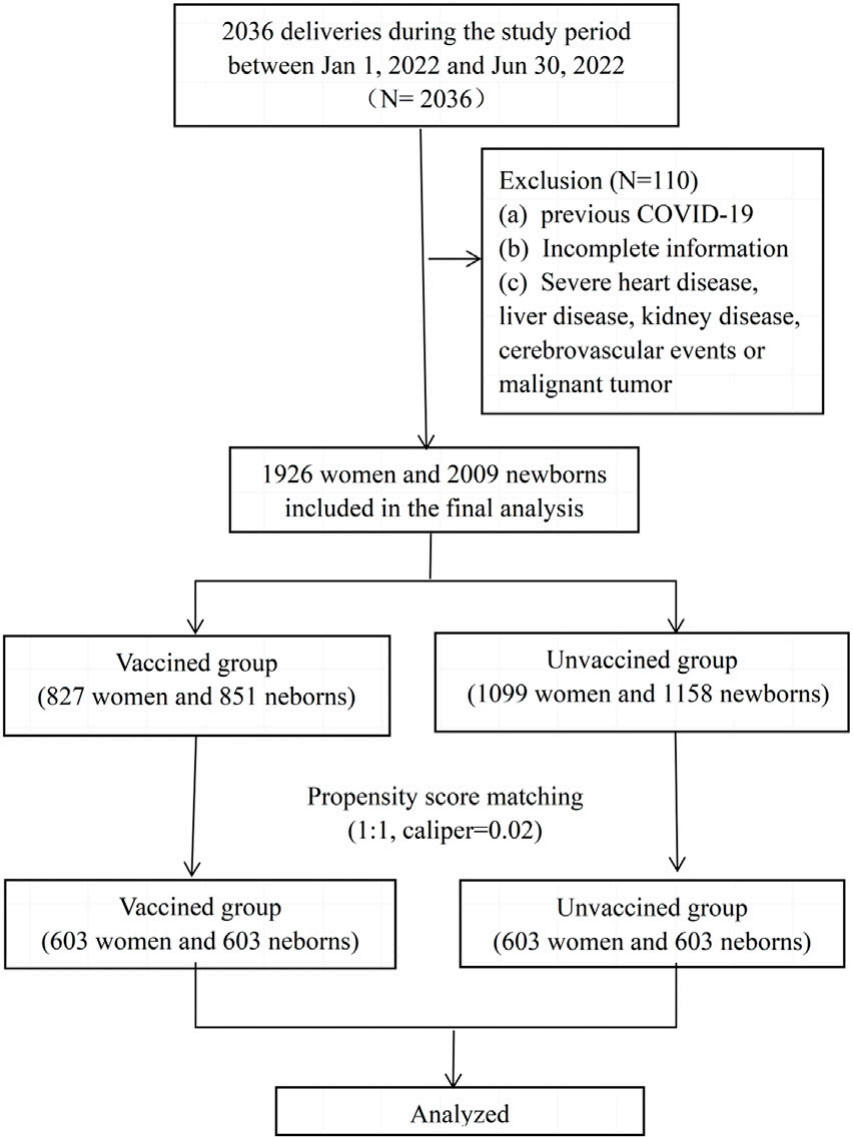
Flow chart of the study.

This was a retrospective cohort study, including all women who deliveries between January and June 2022 at the First Affiliated Hospital of Wenzhou Medical University. Both vaginal and c-section deliveries were included in the study. The study was approved by the Independent Ethics Committee of the First Affiliated Hospital and Wenzhou Medical University (number: KY2023-R038). Exclusion criteria included: (a) ocumented previous positive SARSCoV-2 polymerase chain reaction (PCR) test, (b) incomplete information (unknown vaccination status or pregnancy follow-up information), (c) severe heart disease, liver disease, kidney disease, cerebrovascular events or malignant tumor. Among the enrolled cases, who have completed at least one dose of an inactivated vaccine before or during pregnancy were included in “vaccinated group,” and those who were not vaccinated were included in “unvaccinated group.” All the patients included in this study requested the vaccination voluntarily. The detailed flow chart was shown in Figure 1. Data of demographic characteristics and obstetric and newborn details were extracted from the electronic database. Detailed vaccination status, including the date, dose and manufacturer of the vaccines were recorded in the mobile phone buildin app or accessed through Regional Health Information Resource Center Platform of Zhejiang which were developed by local public health surveillance system.

The primary outcome of the study was the composite adverse pregnancy and maternal outcome, which was defined by one or more of the following: multifetal gestation, caesarean delivery (CD), chorioammonitis, *postpartum* haemorrhage (PPH, estimated blood loss of >1,000 mL), placental abruption, amniotic fluid pollution, premature rupture, polyhydramnios, oligohydramnios, gestational hypertemsion (gHTN, defined as an elevated systolic blood pressure (SBP) of ≥140 mmHg or a diastolic blood pressure (DBP) of ≥90 mmHg occurring on two occasions at least 4 h apart after 20.weeks of gestation, which is not associated with proteinuria and no signs or symptoms of end organ damage), preeclampsia, Gestational diabetes mellitus (GDM, defined as glucose intolerance of variable severity with onset or first recognition during pregnancy), hypothyroidism during pregnancy, placenta previa or placenta implantation. Mode of delivery and length of *postpartum* hospital stay were also assessed.

The secondary outcome was adverse neonatal outcome, which was defined by one or more of the following: preterm birth (PTB, defined as delivery before 37 completed weeks of gestation), very preterm birth (delivery at 28–32 weeks), moderate preterm birth (delivery at 32–33 weeks), late preterm birth (delivery at 34–36 weeks) (World Health Organization. News, 2018), Low birth weight (LBW, birth weight <2,500 g), Very low birth weight, birth weight<1,500 g), Apgar score≤7 at 5-tminute, All-cause hospitalizations, infant death over the study period, jaundice, dirth asphyxia, newborn digestive complications (including neonatal intestinal obstruction, gastrointestinal dysfunction, allergic enteritis, and gastrointestinal bleeding), newborn respiratory complications (including apnea, dyspnea, shortness of breath, respiratory failure, wet lung, pneumonia, and respiratory distress syndrome), newborn fever, neonatal heart disease, sepsis, hypoglycaemia, hyperglycemia, intracranial haemorrhage, newborn feeding problems, aspiration syndrome, composite adverse neonatal outcome (refers to the comprehensive situation of multiple diseases related to premature delivery). Length of newborn hospitalizations, birth weight and gender were also assessed.

## Statistical analysis

We compared the following aspects of the vaccinated group *versus* the unvaccinated group: baseline characteristics, complications during pregnancy, *postpartum* outcomes, and neonatal outcomes. Quantitative variables described by a normal distribution were presented as Mean ± Standard Deviation (M±SD), and were presented as median (interquartile range) if not normally distributed. Differences in quantitative variables between the two groups were compared by Kruskal Wallis test. Categorical variables were presented with n (%) and differences between groups were compared using Chi-square test, and Fisher’s exact test was used if the count variable had a theoretical number <10.

Considering the differences in baseline characteristics between eligible participants in the two groups (Table 1), propensity-score matching (PSM) was used to identify a cohort of patients with similar baseline characteristics. The propensity score was calculated by using a multiple logistic regression model, with vaccinated *versus* unvaccinated serving as the dependent variable, and parity, previous caesarean delivery and fertility treatments serving as independent variables. Matching was performed with the use of a 1:1 matching protocol, with a caliper width of 0.05 of the SD of the logit of the propensity score. SD were estimated for all the baseline covariates before and after matching, SD of less than 10.0% was considered to indicate a balance (Wu et al., 2021). Multivariable logistic regression and multiple linear regression were applied to analyze the influence of COVID-19 vaccination on gestational age at delivery, gestational age at delivery <37 weeks, LBW, VLBW and fetal macrosomia. We used three adjustment models, Model I without any adjustment, Model II adjusted for female age and male age, and Model III adjusted for female age, male age, parity, previous miscarriages, Body Mass Index (BMI), previous caesarean delivery, fertility treatments, pre-existing hypertension.

**TABLE 1:** Baseline characteristics of the vaccinated *versus* the unvaccinated group.

	Before PSM		*p*-Value	After PSM		*p*-Value
Features	Vaccinated (*n* = 827)	Unvaccinated (*n* = 1099)		Vaccinated (*n* = 603)	Unvaccinated (*n* = 603)	
Female Age, years	30.64 ± 4.77	30.52 ± 4.35	0.88	30.58 ± 4.62	30.43 ± 4.29	0.57
Male Age, years	32.67 ± 5.19	32.45 ± 4.77	0.49	32.49 ± 5.04	32.34 ± 4.60	0.59
Parity	2.00 (1.00–4.00)	1.00 (1.00–4.00)	<0.01	2.00 (1.00–4.00)	2.00 (1.00–4.00)	1.00
Previous miscarriages	0.00 (0.00–6.00)	0.00 (0.00–5.00)	0.33	0.00 (0.00–5.00)	0.00 (0.00–5.00)	0.06
BMI, kg/m2	21.46 ± 3.21	21.48 ± 3.19	0.64	21.42 ± 3.14	21.35 ± 3.07	0.70
Previous caesarean delivery, any	178 (21.5%)	178 (16.2%)	<0.01	133 (22.1%)	125 (20.7%)	0.62
Fertility treatments	50 (6.1%)	256 (23.3%)	<0.01	49 (8.1%)	49 (8.1%)	1.00
Pre-existing Hypothyroidism	19 (2.3%)	33 (3.0%)	0.35	80 (13.3%)	88 (14.6%)	0.56
Pre-existing Diabetes	13 (1.6%)	15 (1.4%)	0.71	8 (1.3%)	9 (1.5%)	1.00
Pre-existing hypertension	11 (1.3%)	16 (1.5%)	0.82	9 (1.5%)	4 (0.7%)	0.26
Vaccination during pregnancy	218 (26.4%)	0 (0.0%)	<0.01	0 (0.0%)	0 (0.0%)	NA

In addition, we conducted a sub-analysis in which we divided the vaccinated population into two groups, vaccination before pregnancy group and vaccination during pregnancy group, to compare the differences in maternal and neonatal outcomes between the two groups.

IBM SPSS Statistics 21 (IBM SPSS, Turkey) program and EmpowerStats software (www.empowerstats.com; X&Y solutions, Inc., Boston MA) were used to analyze all the data. In all analyses, *p* < 0.05 were considered statistically significant.

## Results

During the study period, there were 2036 deliveries in the first affiliated hospital of Wenzhou Medical University. A total of 1926 (94.6%) women were enrolled in this study, 827 women were prenatally vaccinated and included in the vaccinated group, and 1099 women were unvaccinated and included in the unvaccinated group. The flow chart of the study was shown in Figure 1. Women in the two groups had similar demographics, with the exception of parity, previous caesarean delivery and the ratio of fertility treatments and the ratio of vaccination during pregnancy (Table 1). We applied the PSM to balance the basic characteristics, and the results showed the baseline and previous obstetric characteristics were well balanced after PSM.

**FIGURE 2:**
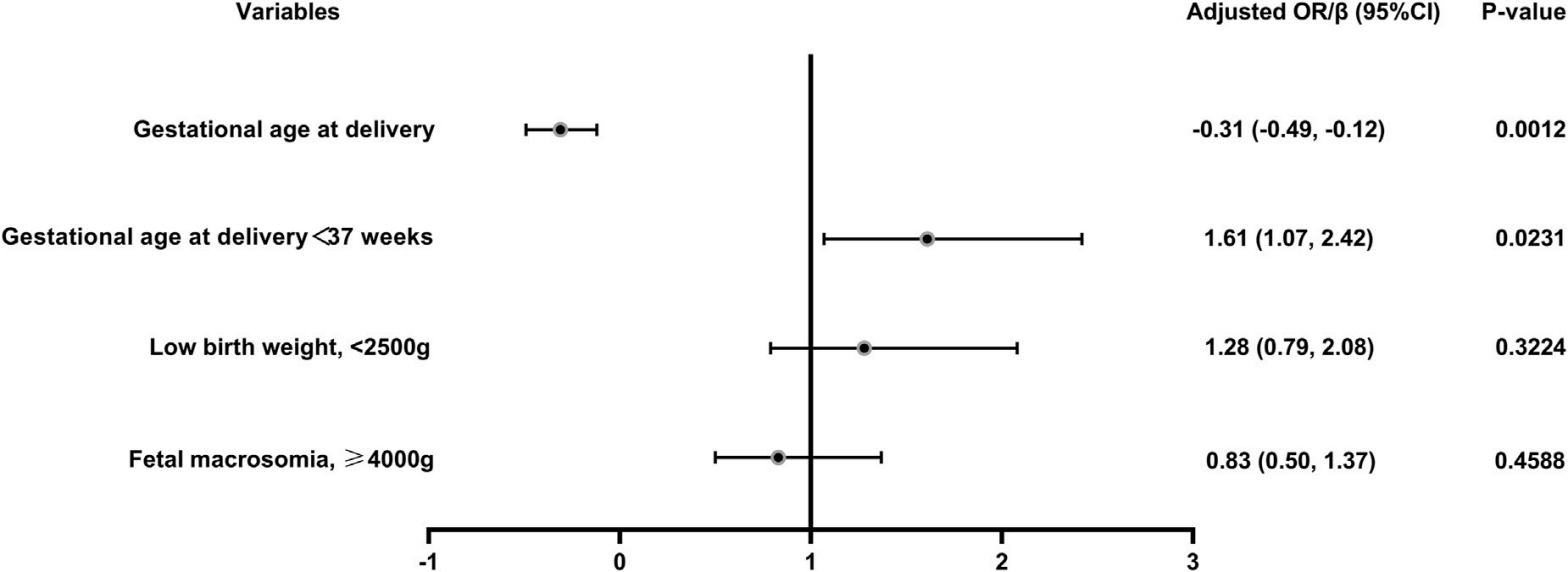
Logistic regression of the maternal and neonatal outcomes in vaccinated women *versus* unvaccinated group. Adjusted for female age, male age, parity, previous miscarriages, BMI, previous caesarean delivery, fertility treatments and pre-existing hypertension; OR, odds ratio.

The pregnancy and maternal outcomes were presented in Table 2. After PSM, the gestational weeks of delivery were slightly lower in the vaccinated group, 38.61 ± 1.89 weeks in the vaccinated group and 38.93 ± 1.49 weeks in the unvaccinated group. Given this, we further analysed the participants who delivered before 37 weeks’ gestation in the two groups and found that there was no significant difference in gestational age at delivery (34.5 ± 2.2 vs. 35.4 ± 1.3, *p* = 0.07). There was a higher rate of overall preterm delivery in the vaccinated group (aOR 1.61, 95% CI 1.07–2.42; *p* = 0.02; Table 3; Figure 2), however, the probability of delivery before 34 weeks and before 32 weeks (very preterm delivery) were similar (*p* > 0.05). The results reveal that there were similar rate of multifetal gestation, abnormal fetal presentation, cesarean section and forceps assisted delivery (*p* > 0.05). There were also similar maternal length of stay (*p* > 0.05) and similar rates of obstetric complications (*p* > 0.05) in the two groups, including *postpartum* hemorrhage, premature rupture of membranes, amniotic fluid contamination, hypoamniotic fluid, gestational hypertension, pre-eclampsia, gestational diabetes mellitus, hypothyroidism in pregnancy, placenta praevia and placental implantation. In addition, there were no diffierence in rate of puerperal infections during pregnancy (*p* > 0.05).

**TABLE 2:** Pregnancy and maternal outcomes of the vaccinated *versus* the unvaccinated group.

	Before PSM		*p*-Value	After PSM		*p*-Value
	Vaccinated (*n* = 827)	Unvaccinated (*n* = 1099)		Vaccinated (*n* = 603)	Unvaccinated (*n* = 603)	
Multifetal gestation	24 (2.9%)	59 (5.4%)	0.01	23 (3.8%)	27 (4.5%)	0.66
Gestational age at delivery	38.74 ± 1.81	38.89 ± 1.57	0.21	38.61 ± 1.89	38.93 ± 1.49	<0.01
Gestational age at delivery<32 weeks	9 (1.1%)	3 (0.3%)	0.02	8 (1.3%)	0 (0.0%)	0.01
Gestational age at delivery<34 weeks	20 (2.4%)	16 (1.5%)	0.12	19 (3.2%)	8 (1.3%)	0.05
Gestational age at delivery<37 weeks	83 (10.0%)	91 (8.3%)	0.18	69 (11.4%)	46 (7.6%)	0.03
Abnormal fetal presentation	29 (3.5%)	53 (4.8%)	0.16	23 (3.8%)	30 (5%)	0.40
Mode of delivery			0.01			0.52
Caesarean delivery	294 (35.6%)	439 (40.0%)	0.05	227 (37.6%)	240 (39.8%)	0.48
Vaginal delivery	512 (61.9%)	615 (56.0%)	0.01	359 (59.5%)	351 (58.2%)	0.68
Forceps assisted delivery	21 (2.5%)	45 (4.1%)	0.06	17 (2.8%)	12 (2%)	0.45
*Postartum* hospital stay	3.73 ± 0.93	3.89 ± 1.28	<0.01	3.75 ± 0.95	3.80 ± 1.01	0.39
Puerperal infection	36 (4.4%)	59 (5.4%)	0.31	24 (4.0%)	23 (3.8%)	1.00
Chorioamnionitis	14 (1.7%)	26 (2.4%)	0.31	9 (1.5%)	4 (0.7%)	0.26
*Postartum* haemorrhage	4 (0.5%)	9 (0.8%)	0.37	3 (0.5%)	9 (1.5%)	0.15
Placental abruption	16 (1.9%)	14 (1.3%)	0.25	7 (1.2%)	8 (1.3%)	1.00
Amniotic fluid pollution	95 (11.5%)	155 (14.1%)	0.09	69 (11.4%)	70 (11.6%)	1.00
Premature rupture of membranes	181 (21.9%)	222 (20.2%)	0.37	135 (22.4%)	125 (20.7%)	0.53
Polyhydramnios	8 (0.9%)	3 (0.3%)	0.05	5 (0.8%)	2 (0.3%)	0.45
Oligohydramnios	47 (5.7%)	78 (7.1%)	0.21	33 (5.5%)	40 (6.6%)	0.47
Gestational hypertemsion	28 (3.4%)	41 (3.7%)	0.69	19 (3.2%)	19 (3.2%)	1.00
Preeclampsia	23 (2.8%)	62 (5.7%)	<0.01	16 (2.7%)	26 (4.3%)	0.16
Gestational diabetes mellitus	129 (15.6%)	213 (19.4%)	0.03	87 (14.5%)	105 (17.4%)	0.16
Hypothyroidism during pregnancy	110 (13.3%)	173 (15.7%)	0.13	17 (2.8%)	14 (2.3%)	0.72
Placenta previa or placenta implantation	2 (0.2%)	9 (0.8%)	0.10	2 (0.3%)	5 (0.8%)	0.45

**TABLE 3:** Multivariate regression analysis for the association between COVID-19 vaccination and preterm birth in the Propensity-Score-Matched Cohort.

Outcome	Model I β/OR (95% CI) *p*-value	Model II β/OR (95% CI) *p*-value	Model III β/OR (95% CI) *p*-value
Gestational age at delivery	−0.32 (−0.51, −0.12) <0.01	−0.31 (−0.50, −0.12) <0.01	−0.31 (−0.49, −0.12) <0.01
Gestational age at delivery<32 weeks	inf. (0.00, Inf) 0.99	inf. (0.00, Inf) 0.99	inf. (0.00, Inf) 0.99
Gestational age at delivery<37 weeks	1.56 (1.06, 2.31) 0.03	1.56 (1.06, 2.31) 0.03	1.61 (1.07, 2.42) 0.02

Model I adjust for: None Model II adjust for: Female age, male age Model III adjust for: Female age, male age, parity, previous miscarriages, BMI, previous caesarean delivery, fertility treatments, pre-existing hypertension.

In this study, a total of 2009 infants were born, 851 in the vaccinated group and 1158 in the unvaccinated group. There were similar neonatal outcomes in the two groups before and after PSM (Table 4). After propensity-score matching, there was no differernce in average weight of newborns, as well as the incidence of macrosomia (aOR = 0.83, 95% CI 0.50–1.37, *p* = 0.46), low birth weight (aOR = 1.28, 95% CI 0.79–2.08, *p* = 0.32) (Table 4; Figure 2). There was also no difference in the sex ratio of newborns (*p* > 0.05). There were similar neonatal mortality, hospitalization rate and length of newborn hospitalization in the two groups (*p* > 0.05). Further detailed data shows that, there were comparable newborn complications in the vaccinated *versus* the unvaccinated group, including the incidence of jaundice, birth asphyxia, newborn digestive complications, newborn respiratory complications, newborn fever, neonatal heart disease, sepsis, hypolycaemia, hyperglycemia, internal haemorrhage, feeding problems of newborn, aspiration syndrome and other composite adverse neonatal outcomes (*p* > 0.05) (Table 4).

**TABLE 4:** Neonatal outcomes of the vaccinated *versus* the unvaccinated group.

	Before PSM		*p*-Value	After PSM		*p*-Value
	Vaccinated (*n* = 851)	Unvaccinated (*n* = 1158)		Vaccinated (*n* = 603)	Unvaccinated (*n* = 603)	
Birth weight, g	3220.35 ± 528.64	3211.16 ± 538.42	0.65	3216.86 ± 532.07	3262.59 ± 493.51	0.21
Birthweight >4000 g	43 (5.1%)	66 (5.7%)	0.53	30 (5.0%)	36 (6.0%)	0.45
Low birth weight, <2500 g	65 (7.6%)	99 (8.6%)	0.46	42 (7.0%)	35 (5.8%)	0.41
Very low birth weight, <1500 g	9 (1.1%)	13 (1.1%)	0.89	8 (1.3%)	2 (0.3%)	0.06
Male gender	447 (52.5%)	599 (51.7%)	0.72	327 (54.2%)	307 (51.0%)	0.25
5-min Apgar score≤7	1 (0.1%)	2 (0.2%)	1.00	1 (0.2%)	0 (0.0%)	1.00
All-cause hospitalizations	210 (24.7%)	328 (28.3%)	0.07	147 (24.4%)	157 (26.0%)	0.51
Length of newborn hospitalization, days	2.17 ± 5.69	2.57 ± 6.37	0.06	2.13 ± 5.86	1.97 ± 4.44	0.48
Infant death over the study period	1 (0.1%)	1 (0.1%)	0.83	1 (0.2%)	0 (0.0%)	0.50
Jaundice	82 (9.6%)	120 (10.4%)	0.59	66 (11.0%)	56 (9.3%)	0.34
Birth asphyxia	7 (0.8%)	8 (0.7%)	0.74	1 (0.2%)	4 (0.7%)	0.37
Newborn digestive complications	3 (0.4%)	9 (0.8%)	0.22	3 (0.5%)	3 (0.5%)	1.00
Newborn respiratory complications	37 (4.4%)	48 (4.2%)	0.82	24 (4.0%)	27 (4.5%)	0.67
Newborn fever	4 (0.5%)	10 (0.9%)	0.30	6 (1.0%)	3 (0.5%)	0.51
Neonatal heart disease	3 (0.4%)	4 (0.4%)	1.00	3 (0.5%)	3 (0.5%)	1.00
Sepsis	1 (0.1%)	6 (0.5%)	0.26	5 (0.8%)	1 (0.2%)	0.22
Hypoglycaemia	5 (0.6%)	8 (0.7%)	0.78	6 (1.0%)	3 (0.5%)	0.51
Hyperglycemia	0 (0.0%)	2 (0.2%)	0.51	1 (0.2%)	0 (0.0%)	1.00
Intracranial haemorrhage	0 (0.0%)	3 (0.3%)	0.27	2 (0.3%)	0 (0.0%)	0.50
Feeding problems of newborn	13 (1.5%)	13 (1.1%)	0.43	6 (1.0%)	10 (1.7%)	0.31
Aspiration syndrome	2 (0.2%)	8 (0.7%)	0.15	4 (0.7%)	1 (0.2%)	0.37
Other composite adverse neonatal outcome	54 (6.4%)	89 (7.7%)	0.25	35 (5.8%)	37 (6.1%)	0.81

In addition, we conducted a sub-analysis in which we divided the vaccinated population into two groups, vaccination before pregnancy (*n* = 609) and vaccination during pregnancy group (*n* = 218), and the results showed that vaccination during pregnancy did not have a significant negative impact on maternal and neonatal outcomes compared with vaccination before pregnancy (Supplementary Tables S1–4).

## Disscussion

To our knowledge, this study is the first to focus on the impact of prenatal inactivated COVID-19 vaccination on maternal and neonatal outcomes. During the study period, 42.9% of women who delivered between January and June 2022 included in the study received at least one dose of vaccine before delivery. In this study, we found no significant association between vaccination and severe adverse pregnancy outcomes or neonatal complications. Although we found a slightly lower gestational week of delivery and a possible increased rate of late preterm birth in the vaccination group, there was no difference in the gestational weeks of pregnant women with preterm births, and no difference in mean neonatal weight, incidence of low birth weight and other neonatal adverse complications.

To date, most studies supported the safety and protection of COVID-19 vaccine in pregnant women and their newborns (Wainstock et al., 2021; Fell et al., 2022; Goldshtein et al., 2022; Rawal et al., 2022). However, most of the studies and evidence were based on mRNA vaccination. A meta-analysis evaluated evidence from 23 studies including 117,552 COVID-19 vaccinated pregnant women, almost exclusively with mRNA vaccines, and the results showed that the mRNA vaccines appear highly effective against SARS-CoV-2 in pregnancy. The incidence of adverse pregnancy outcomes was not increased among vaccinated compared with unvaccinated pregnancies. In fact, surprisingly, the incidence of stillbirths and possibly, preterm births, was lower among the vaccinated pregnant population (Prasad et al., 2022). A multicenter retrospective study conducted in Israel between January and April 2021 demonstrated a similar conclusion, showing that COVID-19 mRNA vaccination (BNT162b2) was not associated with maternal composite adverse outcomes and a significantly lower risk of neonatal composite adverse outcomes was observed (Rottenstreich et al., 2022). In addition, another retrospective cohort study included 4,399 women who delivered between January and June 2021 at Soroka University Medical Center, found no differences in prenatal in terms of pregnancy, delivery, and neonatal complications, including gestational age at delivery, incidence of small for gestational age, and neonatal respiratory complications between women who received COVID-19 mRNA vaccines (BNT162b2) during pregnancy and unvaccinated women (Wainstock et al., 2021). Furthermore, in a population based retrospective cohort study conducted in Canada between May and December 2021, the findings suggested that COVID-19 mRNA vaccination during pregnancy is not associated with a higher risk of preterm birth, small for gestational age at birth, or stillbirth (Fell et al., 2022). In conclusion, none of these vaccines were found to have maternal composite adverse outcomes, nor increased neonatal mortality or respiratory complications. Notably, a retrospective cohort conducted by Aharon Dick et al., 2022 demonstrated a higher risk of preterm delivery in those who were vaccinated with mRNA vaccines in the midterm of pregnancy. However, the extrapolation of these conclusions to inactivated COVID-19 vaccines poses uncertainties.

In China, COVID-19 inactivated vaccine is the main type of COVID-19 vaccine available (Fu et al., 2022). Actually, inactivated COVID-19 vaccines were account for almost half of all doses delivered globally, and had been tremendously crucial in fighting the pandemic (Mallapaty, 2021a; Mallapaty, 2021b). Studies have shown that COVID-19 inactivated vaccines are effective in preventing the transmission of COVID-19, and reducing the risk of symptomatic COVID-19 with few serious adverse events (Al Kaabi et al., 2021; Jara et al., 2021). An animal study showed that human angiotensin-converting enzyme 2 (hACE2) mice vaccinated with inactivated COVID-19 vaccine before and during pregnancy exhibited normal body weight changes and reproductive performance indicators, and the physical development of their offspring was normal. Moreover, after intranasal vaccination with inactivated COVID-19 vaccine, all pregnant mice in the immunized group survived, and the reproductive performance and physical development of their offspring were normal. In contrast, all mice in the non-immunized group died before delivery. This study may indicate that inactivated COVID-19 vaccination is safe and may effectively protects pregnant mice from COVID-19 and has no adverse effects on offspring (Lin et al., 2022). Huang et al., 2022 demonstrates that female vaccination with inactivated COVID-19 vaccine does not have any measurable deleterious effects on *in vitro* fertilization treatment and has no significant impact on embryonic laboratory parameters or pregnancy outcomes. Another study on the prognosis of inactivated COVID-19 vaccine in frozen-thawed embryos for transfer revealed that live birth rates, sustained pregnancy rates and clinical pregnancy rates in vaccinated women were comparable to those in unvaccinated women, and birth length and birth weight were similar in both groups (Cao et al., 2022). Consistent with our findings, these studies did not find any adverse effects of vaccination on newborns. However, none of these studies reported maternal-related complications, and only birth weight was analyzed regarding neonatal outcomes. The present study is the first to focus on the effects of prenatal inactivated COVID-19 vaccination on maternal and neonatal outcomes and to analyze the relevant outcomes in details, with results largely similar to the previous studies. However, we found an increased risk of overall preterm birth in the vaccinated group compared to unvaccinated women (*p* < 0.05), which we think may be due to the fact that the present study was retrospective, and although we adjusted for all the variables as much as possible, there may still be potential unadjusted bias that could have contributed to this result. And what needs to be declared is that there was no difference in very preterm birth (<32 weeks) rate and incidence of preterm birth<34 weeks in our study. Interestingly, we found that although the rate of preterm birth may have increased, there was no difference in the gestational weeks of pregnant women with preterm births, which may explain why there was no difference in the birth weight of the newborns and the risk of other adverse birth outcomes.

In this study, we have analysed pregnancy and neonatal outcomes in great detail, and we also used a matched cohort of propensity scorebalanced women to control for potential confounders. This study presents several novel discoveries but still has some limitations. Firstly, due to the single-center retrospective nature of the study, some baseline clinical characteristics may not have been adequately balanced. Second, due to the limited sample size, we were unable to match by time of the first vaccination. In addition, early pregnancy outcomes such as miscarriage and ectopic pregnancy cannot be studied.

## Conclusion

In this cohort study, the inactivated COVID-19 vaccine appeared to be safe, and although there was an increased incidence of late preterm delivery compared with unvaccinated women, there was no significant increase in other adverse maternal and neonatal outcomes. Future studies that focus on outcomes at the time of vaccination may provide better understanding and more accurate counseling of patients regarding the risks and benefits of vaccination before and during pregnancy.

## Data Availability

The original contributions presented in the study are included in the article/Supplementary Material, further inquiries can be directed to the corresponding author.
